# Complete blood count in acute kidney injury prediction: a narrative review

**DOI:** 10.1186/s13613-019-0561-4

**Published:** 2019-08-06

**Authors:** Joana Gameiro, José António Lopes

**Affiliations:** Division of Nephrology and Renal Transplantation, Department of Medicine, Centro Hospitalar Lisboa Norte, EPE, Av. Prof. Egas Moniz, 1649-035 Lisbon, Portugal

**Keywords:** Acute kidney injury, Prognosis, Epidemiology, Biomarkers, Complete blood count, Ratio

## Abstract

Acute kidney injury (AKI) is a complex syndrome defined by a decrease in renal function. The incidence of AKI has raised in the past decades, and it is associated with negative impact in patient outcomes in the short and long term. Considering the impact of AKI on patient prognosis, research has focused on methods to assess patients at risk for developing AKI, diagnose subclinical AKI, and on prevention and treatment strategies, for which it is crucial an understanding of pathophysiology the of AKI. In this review, we discuss the use of easily available parameters found in a complete blood count to detect patients at risk for developing AKI, to provide an early diagnosis of AKI, and to predict associated patient outcomes.

## Background

Acute kidney injury (AKI) is a complex syndrome defined as a rapid decrease in renal function, caused by numerous etiologies [1]. The incidence of AKI has raised in the past decades, occurring in up to 20% of hospitalized patients and the incidence is higher in critically ill patients [2, 3].

AKI has a negative impact in patient outcomes in the short and long term, increasing the risk of in-hospital mortality, longer hospital stays, cardiovascular events, progression to chronic kidney disease, and long-term mortality [4–6]. Overall, mortality rates associated with AKI have decreased reflecting the impact of the increased recognition of this diagnosis and improvements in patient care. Nevertheless, mortality rates are significant and increase with AKI severity, specifically in dialysis-requiring AKI [7, 8].

Given its impact on prognosis, it is important to recognize risk factors for its occurrence, such as advanced age, diabetes, hypertension, chronic kidney disease (CKD), cardiovascular disease (CVD), liver disease, lung disease, disease severity, sepsis, shock, nephrotoxicity, and surgery [7–10] (Table 1).

**Table 1:** Risk factors for AKI

AKI risk factors
Advanced age
Diabetes
Hypertension
Chronic kidney disease
Cardiovascular disease
Liver disease
Lung disease
Disease severity
Shock
Sepsis
Nephrotoxins
Surgery

In the past decade, numerous studies have increased the understanding of the pathophysiology of AKI which led to the investigation of novel biomarkers. Nevertheless, the use of these biomarkers is limited in clinical practice.

The purpose of this review is to discuss the use of easily available parameters found in a complete blood count to detect patients at risk for developing AKI and to predict associated patient outcomes.

## AKI definition and biomarkers

The definition of AKI has evolved significantly since 2004 when the Risk Injury Failure Loss of kidney function End-stage kidney disease (RIFLE) classification was first published [11]. The Acute Kidney Injury Network (AKIN) classification further revised these criteria, improving its diagnostic sensitivity and specificity [7]. The current definition of AKI was proposed by the Kidney Disease Improving Global Outcomes (KDIGO) work group in 2012 [12]. The KDIGO classification defines AKI as an increase in serum creatinine (SCr) of at least 0.3 mg/dl within 48 h, or an increase in SCr to more than 1.5 times baseline which is known or presumed to have occurred within the prior 7 days, or a urine output (UO) decrease to less than 0.5 ml/kg/h for 6 h. This classification also stratifies AKI in stages of severity which correlate with patient prognosis [12] (Table 2).

**Table 2:** Risk, injury, failure, loss of kidney function, End-stage kidney disease (RIFLE) [11], Acute Kidney Injury Network (AKIN) [7], and Kidney Disease Improving Global Outcomes (KDIGO) [12] classifications

Class/stage	SCr/GFR			UO		
	RIFLE	AKIN	KDIGO	RIFLE	AKIN	KDIGO
Risk/1^a^	↑ SCr X 1.5 or ↓ GFR > 25%	↑ SCr ≥ 26.5 μmol/l (≥ 0.3 mg/dl) or ↑ SCr ≥ 150 to 200% (1.5 to 2X)	↑ SCr ≥ 26.5 μmol/l (≥ 0.3 mg/dl) or ↑ SCr ≥ 150 to 200% (1.5 to 2X)	< 0.5 ml/kg/h (> 6 h)	< 0.5 ml/kg/h (> 6 h)	< 0.5 ml/kg/h (> 6 h)
Injury/2^a^	↑ SCr X 2 or ↓ GFR > 50%	↑ SCr > 200 to 300% (> 2 to 3X)	↑ SCr > 200 to 300% (> 2 to 3X)	< 0.5 ml/kg/h (> 12 h)	< 0.5 ml/kg/h (> 12 h)	< 0.5 ml/kg/h (> 12 h)
Failure/3^a^	↑ SCr X 3 or ↓ GFR > 75% or if baseline SCr ≥ 353.6 μmol/l (≥4 mg/dl) ↑ SCr > 44.2 μmol/l (> 0.5 mg/dl)	↑ SCr > 300% (> 3X) or if baseline SCr ≥ 353.6 μmol/l (≥ 4 mg/dl) ↑SCr ≥ 44.2 μmol/l (≥ 0.5 mg/dl) or initiation of renal replacement therapy	↑ SCr > 300% (> 3X) or ↑SCr to ≥ 353.6 μmol/l (≥ 4 mg/dl) or initiation of renal replacement therapy	< 0.3 ml/kg/h (> 24 h) or anuria (> 12 h)	< 0.3 ml/kg/h (24 h) or anuria (12 h)	< 0.3 ml/kg/h (24 h) or anuria (12 h)

*SCr* serum creatinine, *GFR* glomerular filtration rate, *UO* urine output, *RIFLE* Risk, Injury, Failure, Loss of kidney function (dialysis dependence for at least 4 weeks), End-stage kidney disease (dialysis dependence for at least 3 months), *AKIN* Acute Kidney Injury Network, *KDIGO* Kidney Disease Improving Global Outcomes

^a^Risk class (RIFLE) corresponds to stage 1 (AKIN and KDIGO), Injury class (RIFLE) corresponds to stage 2 (AKIN and KDIGO), and Failure class (RIFLE) corresponds to stage 3 (AKIN and KDIGO)

The definition of AKI depends on SCr and UO, which are imperfect markers of AKI [13–15]. SCr is an insensitive and delayed marker which is altered by factors affecting its production (age, gender, diet, and muscle mass), elimination (previous renal dysfunction), and secretion (medications). UO depends on patient’s volemic and haemodynamic status and use of diuretics, is difficult to assess without a urinary catheter and its usefulness relies on an hourly assessment which is time-consuming [13–17].

Therefore, research has focused on the development of new biomarkers, including Cystatin-C, neutrophil gelatinase-associated lipocalin (NGAL), *N*-acetyl-glucosaminidase (NAG), kidney injury molecule 1 (KIM-1), interleukin-6 (IL-6), interleukin-8 (IL-8), interleukin 18 (IL-18), liver-type fatty acid-binding protein (L-FABP), calprotectin, urine angiotensinogen (AGT), urine microRNAs, insulin-like growth factor-binding protein 7 (IGFBP7), and tissue inhibitor of metalloproteinases-2 (TIMP-2) [18–20]. These have been evaluated and validated in multiple settings, though their use in clinical practice is limited due to the associated costs and lack of evidence of better patient outcomes with their use in detecting AKI [20, 21].

A comprehensive understanding of the pathophysiology is critical to identify easily available predictors of AKI to prevent, diagnose, and treat this complication, ultimately improving patient outcomes.

## Pathophysiology of AKI

AKI can be systematized clinically into three groups: prerenal, which accounts for up to 60% of cases and results from the functional adaptation to hypoperfusion of structurally normal kidneys; intrinsic renal results from structural damage to any component of the renal parenchyma and accounts for up to 40% of cases; and less frequently postrenal resulting from urinary tract obstruction [22–24].

Most cases are multifactorial and following the inciting event numerous pathophysiologic events occur, including hemodynamic instability, microcirculatory dysfunction, tubular cell injury, tubular obstruction, renal congestion, microvascular thrombi, endothelial dysfunction, and inflammation [23–26]. In recent years, the significant role of inflammation in the pathophysiology of AKI has been emphasized [27].

The immunopathogenesis of AKI involves a complex interaction of damage-associated molecular patterns, pathogen-associated molecular patterns, oxidative stress, hypoxia inducible factor, complement system, dendritic cells, neutrophils, lymphocytes, macrophages, platelets, and cytokines [28–31].

The initial damage to renal endothelial cells and proximal tubular epithelial cells produces cytokines resulting in infiltration of inflammatory cells, such as neutrophils, lymphocytes, and macrophages into the interstitium [24, 26–28]. These cells cause ischemic tubular epithelial and endothelial injury directly and indirectly by releasing oxygen radicals, prostaglandins, leukotrienes, and thromboxanes [28, 29]. In addition, they produce pro and anti-inflammatory cytokines that further increase or decrease inflammation in the kidney [28–30].

Local and systemic inflammation plays a significant part in the initiation and extension phases of AKI, in the multi-organ failure associated with AKI and also in the progression to chronic kidney disease which can result if these immune mechanisms persist [28, 30]. The role of immune cells in the pathogenesis of AKI has been highlighted in several animal models, in which direct or indirect inhibition of immune cells attenuated renal injury [31–34].

Neutrophils are important components of innate immunity and respond rapidly to injury. Their contribution to AKI pathophysiology results from adherence to endothelium and release of cytokines, reactive oxygen species, and proteases [30, 35]. Neutrophils regulate the acute phase of inflammation within the first 24 h, which motivated their role as an early marker of the severity of AKI [36, 37].

Lymphocytes are the main components of adaptive immunity, which also play a significant role in the development and maintenance of AKI, directly by cellular damage and indirectly by producing pro-inflammatory cytokines [30–38]. Natural killer T cells are presumed to induce renal injury as they are directly cytotoxic and release pro-inflammatory cytokines and activate macrophages and neutrophils [39]. CD4+ T cells also contribute to the early phase of AKI. In later phases of AKI, infiltration of lymphocytes and macrophages predominates over neutrophils [40, 41]. T cells appear to have a significant role in the repair phase of AKI and possibly contribute to the transition from AKI to chronic kidney disease. Regulatory T cells are significant in renoprotection and renal regeneration processes, through anti-inflammatory cytokine release and promoting tubular proliferation [42]. Understanding these inflammatory processes may provide insight into future interventions to prevent or attenuate the consequences of AKI.

In addition, platelet and leucocyte interactions appear to be a critical step in inflammation, as both innate and adaptive immunity are mediated by the interaction of neutrophils, monocytes, lymphocytes, and platelets [43–45]. Platelets adhere to the endothelial wall, modulate vascular permeability, recruit and activate leucocytes, and activate the complement system, thus contributing significantly to hemodynamic and inflammatory processes of AKI [45, 46].

Investigation has long focused on quantifying inflammation and determining its prognostic impact on AKI. With the limitations of the contemporary markers of AKI and the prognostic impact of this diagnosis, it was important to evaluate the role of parameters found in a complete blood count as early markers of AKI, disease severity and prognosis. In the following section, we present a review of the studies focused on the correlation of complete blood count results, AKI, and outcomes (Table 3).

**Table 3:** Complete blood count parameters, AKI incidence, and outcomes

Study	Design	Setting	*N*	AKI definition	Incidence of AKI	Mortality
Anemia						
Shema-Didi et al. [50]	Retrospective	Hospitalized	34,802	RIFLE	OR 1.5 (1.4–1.6), *p* < 0.001	OR 6.3 (4.6–8.5), *p* < 0.001
Karkouti et al. [56]	Retrospective	Post-cardiac surgery	1444	SCr ≥ 50%	RR 2.6 (2.0–3.3)	
Shacham et al. [51]	Retrospective	Post-acute myocardial infarction	1248	AKIN	OR 1.76 (1.02–3.02), *p* = 0.04	
Han et al. [53]	Retrospective	Critically ill	2145	KDIGO	OR 1.76 (1.349–2.291), *p* < 0.001	OR 2.21 (1.835–2.662), *p* < 0.001
Arai et al. [59]	Prospective	Post-transcatheter aortic valve implantation	3472	Valve Academic Research Consortium criteria	OR 1.82 (1.45–2.29), *p* < 0.01	HR 1.5 (1.29–1.73), *p* < 0.01
Powell-Tuck et al. [54]	Retrospective	Critically ill	210	AKIN	OR 0.82 (0.65–1.03), *p* = 0.09	
Abusaada et al. [52]	Prospective	Post-acute myocardial infarction	1107	AKIN	OR 1.66 (1.07–2.54), *p* = 0.022	
Choi et al. [61]	Retrospective	Post-total hip replacement arthroplasty	2467	AKIN	OR 2.036 (1.369–3.028), *p* < 0.001	
Malhotra et al. [55]	Prospective	Critically ill	573	KDIGO	OR 1.477 (0.891–2.449), *p* = 0.13	
Duque-Sosa et al. [57]	Retrospective	Post-cardiac surgery	966	KDIGO	OR 1.32 (1.00–1.75), *p* = 0.05	
Gorla et al. [60]	Retrospective	Post-thoracic endovascular aortic repair	144	AKIN	OR 4.34 (1.91–9.85), *p* < 0.001	OR 1.67 (1.03–2.68), *p* = 0.036
Oprea et al. [58]	Retrospective	Post-cardiac surgery	6130	KDIGO	HR 1.15 (1.01 to 1.32), *p* = 0.04	HR 1.50 (1.25 to 1.79), *p* < 0.001
Sreenivasan et al. [62]	Retrospective	Post-coronary angiography	2055	KDIGO	OR 5.3 (3.8–7.3), *p* < 0.001	
White blood cell count						
Han et al. [63]	Prospective	Critically ill	2079	KDIGO	*Leucocytosis* OR 2.05 (1.39–3.031), *p* < 0.001 *Leucopenia* OR 1.42 (1.002–1.996), *p* = 0.048	*Leucocytosis* OR 1.36 (0.992–1.852), *p* = 0.056 *Leucopenia* OR 1.40 (1.025–1.912), *p* = 0.035
Delta-neutrophil index						
Kim et al. [64]	Retrospective	Sepsis-associated AKI	346	AKIN	OR 1.060, *p* < 0.001	HR 25.2, *p* < 0.001
Neutrophil-to-lymphocyte ratio						
Kim et al. [65]	Retrospective	Post-cardiovascular surgery	590	KDIGO	*NLR *>* 4.5* OR 3.26 (1.51–7.06), *p* = 0.003	HR 1.02 (1.01–1.04), *p* = 0.006
Yilmaz et al. [69]	Retrospective	Septic patients	118	AKIN	OR 3.25 (2.72–4.19), *p* < 0.001	
Yuan et al. [68]	Retrospective	Post-coronary angiography	1162	SCr ≥ 0.5 within 3 days	OR 1.105 (1.044–1.169), *p* = 0.001	
Alfeilat et al. [72]	Prospective	Emergency department	294	AKIN/KDIGO	*NLR *>* 5.5* OR 6.4 (2.7–16), *p* = 0.031	
Gameiro et al. [73]	Retrospective	Cirrhotic patients	186	SCr ≥ 0.3 in 48 h or SCr ≥ 50%	OR 1.1 (1.0–1.1), *p* = 0.028; *NLR *> *6* OR 2.4 (1.0–5.8), *p* = 0.041	
Parlar et al. [66]	Retrospective	Post-cardiac surgery	311	KDIGO	OR 0.45 (0.22–0.95), *p* = 0.04	
Bu et al. [70]	Retrospective	Septic patients	132	KDIGO	OR 1.047 (1.005–1.091), *p* = 0.026	
Kim et al. [67]	Retrospective	Burn-injured patients	473	KDIGO	OR 1.094 (1.064–1.125), *p* < 0.001	
Younan et al. [74]	Retrospective	Critically ill trauma	207	KDIGO	OR 2.06 (1.04–4.06), *p* = 0.04	
Fan et al. [75]	Retrospective	Critically ill AKI patients	13,678	KDIGO		*NLR *>* 12.14* HR 1.83 (1.66–2.02), *p* < 0.0001
Mean platelet volume						
Han et al. [76]	Retrospective	Critically ill AKI–CRRT patients	349	Clinical evaluation		HR 1.08 (1.010–1.155), *p* = 0.023
Mean platelet ratio						
Li et al. [78]	Retrospective	Critically ill AKI–CRRT patients	223	KDIGO		AUROC 0.636 (0.563–0.708), *p* < 0.001
Thrombocytopenia						
Van Linden et al. [81]	Retrospective	Post-trans-apical aortic valve implantation	270	RIFLE	OR 4.4 (1.6–12.2), *p* = 0.005	
Fan et al. [83]	Retrospective	Heat stroke patients	176	Clinical evaluation	OR 37.92 (2.18–87.21), *p* < 0.01	
Kertai et al. [82]	Retrospective	Post-cardiac surgery	4217	KDIGO	OR 1.14 (1.09–1.20), *p* < 0.0001	HR 5.46 (3.79–7.89), *p* < 0.0001
Chao et al. [84]	Prospective	Emergency department, elderly	136	KDIGO	HR 1.85 (1.05–3.28), *p* = 0.03	
Wu et al. [85]	Retrospective	Hemorrhagic shock patients	84	KDIGO	OR 0.71 (0.54–0.93), *p* < 0.05	HR 0.89 (0.76–0.99), *p* < 0.05
Neutrophil, lymphocyte and platelet ratio						
Gameiro et al. [87]	Retrospective	Post-abdominal surgery	450	KDIGO	OR 1.05 (1.00–1.10), *p* = 0.048	
Koo et al. [86]	Retrospective	Post-cardiac surgery	1099	KDIGO	OR 1.19 (1.04–1.36), *p* = 0.0124; *NLPR *≥* 64* OR 2.18 (1.20–3.98), *p* = 0.011	*NLPR *>* 3* HR 3.54 (2.00–6.28), *p* < 0.001
Platelet-to-lymphocyte ratio						
Hudzik et al. [89]	Retrospective	Post-acute myocardial infarction, diabetic patients	719	KDIGO	OR 1.22 (1.10–1.34), *p* < 0.0001	
Zheng et al. [91]	Retrospective	Critically ill AKI patients	10,859	KDIGO		*PLR *<* 90* HR 1.25 (1.12–1.39), *p* < 0.001 *PLR *>* 311* HR 1.19 (1.08–1.31), *p* < 0.001
Sun et al. [90]	Retrospective	Post-acute myocardial infarction, coronary angiography	5719	SCr > 25% or > 0.5 mg/dl within 72 h of contrast exposure	OR 1.432 (1.205–1.816,) *p* = 0.031	
Parlar et al. [66]	Retrospective	Post-cardiac surgery	311	KDIGO	OR 1.06 (1.01–1.10), *p* = 0.01	

*AKIN* Acute Kidney Injury Network; *AKI* Acute kidney injury; *AUROC* Area Under the Receiver Operating Characteristics; *GFR* glomerular filtration rate; *HR* Hazard Ratio; *KDIGO* Kidney Disease Improving Global Outcomes; *NLR* neutrophil-to-lymphocyte ratio; *NLPR* neutrophil, lymphocyte, and platelet ratio; *OR* odds ratio; *PLR* platelet-to-lymphocyte ratio; *RIFLE* Risk, Injury, Failure, Loss of kidney function, End-stage kidney disease; *RR* relative risk; *SCr* serum creatinine

## Complete blood count parameters and AKI

### Anemia

Several studies have demonstrated the association of anemia, which is frequent in hospitalized patients and associated with worse outcomes, and AKI [47]. The contributory effects of anemia to AKI are likely multifactorial. The presence of lower hemoglobin predisposes patients to renal hypoxia and oxidative stress [48]. In addition, many anemic patients have subclinical renal disease which increases the susceptibility to renal insults [49].

Shema-Didi et al. reported an independent association between preexisting anemia and in-hospital AKI occurrence (OR 1.5 (1.4–1.6), *p* < 0.001) and that the severity of anemia correlated with AKI requiring renal replacement therapy (RRT) and mortality, in a retrospective study of 34,802 hospitalized patients [50].

In a retrospective cohort of 1248 post-acute myocardial infarction patients, admission hemoglobin levels and anemia were independently associated with AKI (OR 1.76 (1.02–3.02), *p* = 0.04) [51]. Abusaada et al. developed a score to predict AKI risk in patients with acute myocardial infarction based on clinical and laboratory data at admission, including anemia as an independent risk predictor along with decompensated heart failure, baseline renal function, diabetes, and tachycardia [52].

Han et al. identified a hemoglobin threshold for detecting an increased risk of AKI and an association of anemia and AKI and long-term mortality in a retrospective study of critically ill patients [53]. Interestingly, a retrospective study by Powell-Tuck et al. of 210 critically ill patients with AKI stage I diagnosed by the AKI Network (AKIN) classification criteria reported that anemia did not increase the risk to progress to AKI stage III [54]. Malhotra et al. recently developed a risk prediction score in a prospective study of critically ill patients which included anemia as an independent predictor of AKI [OR 1.477 (0.891–2.449), *p* = 0.13] [55].

In cardiac surgery patients, Karkouti et al. reported that the presence of preoperative anemia, intraoperative anemia, and red blood cell transfusion on the day of surgery had a 2.6-fold increase in the relative risk of AKI [56]. A Spanish multicenter retrospective cohort of cardiac surgery patients demonstrated that intraoperative anemia was independently associated with increased risk of AKI [OR 1.32 (1.00–1.75), *p* = 0.05] [57].

Oprea et al. also demonstrated the association of preoperative and postoperative anemia and AKI and AKI severity after coronary artery bypass grafting surgery (HR 1.15 (1.01–1.32), *p* = 0.04). Furthermore, this study also demonstrated an association between anemia and long-term mortality (HR 1.50 (1.25 to 1.79), *p* < 0.001) [58].

The presence of preoperative and postoperative anemia was also independently associated with AKI [OR 1.82 (1.45–2.29), *p* < 0.01] and 1-year mortality [HR 1.5 (1.29–1.73), *p* < 0.01] in a prospective study of patients undergoing transcatheter aortic valve implantation [59].

In patients undergoing thoracic endovascular aortic repair, Gorla et al. identified preoperative hemoglobin levels and postoperative hemoglobin levels decrease as risk factors for AKI and in-hospital mortality [60].

In a cohort of 2467 patients submitted to total hip replacement arthroplasty, postoperative anemia was associated with postoperative AKI [OR 2.036 (1.369–3.028), *p* < 0.001] [61]. Anemia was also an independent predictor of contrast-induced AKI in patients undergoing coronary angiography [62].

Therefore, anemia has long been a marker of poor patient prognosis and is important to take into account when evaluating a patient.

### White blood count

The role of white blood cells (WBC) in the pathophysiology of AKI has been described above. Han et al. demonstrated a U-shaped relationship between WBC count and risk of AKI and mortality in a prospective cohort of critically ill patients [63]. They propose that the higher risk of AKI in leucocytosis could be related to neutrophilia and associated pro-inflammatory function, and the higher risk of AKI in leucopenia could be related to lymphopenia and monocytopenia which result in a lack of protective function [63].

Recently, Kim et al. reported the use of a calculated delta-neutrophil index (DNI), which reflects the fraction of immature WBC, to predict sepsis-associated AKI in the emergency department [64]. In their study, DNI ≥ 14.0% was an independent predictor of severe AKI (OR 7.238, *p*  <  0.001) and severe AKI was an independent predictor of 30-day mortality (HR 25.2, *p*  <  0.001) in a cohort of septic patients [64].

### Neutrophil-to-lymphocyte ratio

The neutrophil–lymphocyte ratio (NLR) is an easily calculated marker of systemic inflammation that has been recently demonstrated to effectively predict AKI in multiple settings.

In a retrospective cohort of 590 patients undergoing cardiovascular surgery, postoperative elevated NLR was significantly associated with an increased risk of postoperative AKI and mortality [65]. Parlar et al. also demonstrated that an increased NLR was associated with postoperative AKI in patients undergoing cardiovascular surgery with cardiopulmonary bypass [66]. An elevated preoperative NLR was also documented as a predictor of AKI in burn surgery patients, in whom a cut-off value of 11.7 was optimal for postoperative AKI prediction (OR 1.094 (1.064–1.125), *p* < 0.001) [67].

The NLR was associated with an increased risk of contrast-induced AKI, defined as an increase in SCr of 0.5 mg/dl within 3 days, in 1162 patients submitted to an emergency percutaneous coronary intervention (OR 1.105 (1.044–1.169), *p* = 0.001) [68].

In patients with sepsis, NLR at admission has been demonstrated as an important predictor of AKI in two retrospective cohorts [69, 70]. Indeed, the systemic inflammation associated with septic-AKI is vital in the development of multi-organ failure [71]. In both studies, there was no correlation between NLR and mortality [71, 72].

Alfeilat et al. reported that a cut-off value of NLR > 5.5 could be used to early detect AKI in a prospective study of 294 patients at emergency department admission (NLR > 5.5 OR 6.4 (2.7–16), *p* = 0.031) [72].

In a cohort of cirrhotic patients, we have developed a risk score for AKI combining renal, liver and inflammatory markers, which included the NLR [73]. In this retrospective study, a higher NLR was independently associated with AKI (13.9 ± 16.5 vs 5.5 ± 4.0, *p* < 0.001; unadjusted OR 1.2 (1.1–1.3), *p* < 0.001; adjusted OR 1.1 (1.0–1.1), *p* = 0.028; NLR > 6 OR 2.4 (1.0–5.8), *p* = 0.041) [73].

Furthermore, an increasing trajectory of NLR over the first 48 h of admission was associated with development of organ failure in critically ill male trauma patients (OR 2.06 (1.04–4.06), *p* = 0.04) [74].

In a retrospective study of 13,678 critically ill AKI patients, Fan et al. demonstrated that an NLR higher than 12.14 was a predictor of all-cause mortality (HR 1.83 (1.66–2.02), *p* < 0.0001) [75].

Although a standardized cut-off value for the NLR has not been defined, this easily calculated marker could be promising in the early diagnosis of AKI and as a prediction of worse outcomes.

### Platelet volume

As described above, platelets have a significant role in the hemodynamic and inflammatory mechanisms of AKI. Thus, several studies have focused on platelet parameters as predictors of AKI and outcomes.

Han et al. demonstrated that mean platelet volume (MPV) ≥ 10.2 fL was a significant prognostic risk factor for 28-day mortality in a retrospective analysis of 349 AKI patients requiring continuous renal replacement therapy (CRRT) (HR 1.08 (1.010–1.155), *p* = 0.023) [76]. An increased MPV reflects increased platelet activity and turnover, which could reflect more severe inflammation and a risk factor for overall vascular mortality [77]. Li et al. developed the mean platelet volume/platelet count ratio (MPR) from a retrospective cohort of critically ill AKI patients CRRT [78]. In this study, MPR > 0.099 was a significant predictor of mortality [AUROC 0.636 (0.563–0.708), *p* < 0.001] [78].

### Thrombocytopenia

Thrombocytopenia has often been reported as an indicator of underlying disease severity and worse patient outcomes [79, 80].

Thrombocytopenia was an independent risk factor for postoperative AKI in patients undergoing minimally invasive transapical aortic valve implantation (OR 4.4 (1.6–12.2), *p* = 0.005) [81]. There was also a significant association between postoperative thrombocytopenia and postoperative AKI (OR 1.14 (1.09–1.20), *p* < 0.0001) and mortality (HR 5.46 (3.79–7.89), *p* < 0.0001), in a retrospective study of patients undergoing after coronary artery bypass grafting surgery [82].

In a retrospective study of patients with heat stroke, thrombocytopenia at admission was also a predictor of AKI (OR 37.92 (2.18–87.21), *p* < 0.01) [83]. In addition, thrombocytopenia was associated with a higher risk of AKI in elderly patients in a prospective study of patients admitted to the emergency department [84].

In patients with hemorrhagic shock, thrombocytopenia in the first 48 h was associated with higher AKI and mortality risk [85]. Moreover, the severity of platelet count decrease had a significant correlation to the severity of AKI and mortality [85].

Hence, it is important to consider thrombocytopenia as an indicator of disease severity when assessing patients with AKI.

### Neutrophil, lymphocyte, and platelet ratio

Koo et al. developed the neutrophil, lymphocyte, and platelet ratio (NLPR) in the setting of AKI after cardiovascular surgery [86]. The NLPR is calculated as follows:

$$({\text{Neutrophil count}} \times 100)/({\text{lymphocyte count}} \times {\text{platelet count}}).$$

In their retrospective study of 1099 patients, higher perioperative NLPR were associated with postoperative AKI (NLPR ≥ 64 OR 2.18 (1.20–3.98), *p* = 0.011) and 5-year mortality (NLPR > 3 HR 3.54 (2.00–6.28), *p* < 0.001). This study demonstrated that adding platelet count to the NLR improved the predictive efficacy when compared to NLR or thrombocytopenia alone [86].

We have conducted a retrospective analysis in a cohort of patients undergoing major abdominal surgery and confirmed the predictive ability of NLPR in detecting AKI in this setting [OR 1.05 (1.00–1.10), *p* = 0.048]; however, in our study, NLPR did not predict mortality [87].

Further studies should be conducted to validate the use of this ratio as a marker to detect risk of AKI and mortality.

### Platelet-to-lymphocyte ratio

In recent publications, platelet-to-lymphocyte ratio (PLR) has been reported as new poor prognostic marker [88].

Hudzik et al. reported an association between higher PLR and risk of developing contrast-induced AKI in a retrospective analysis of diabetic patients with ST-elevation myocardial infarction (OR 1.22 (1.10–1.34), *p* < 0.0001) [89]. In this study, a PLR higher than 110 had a d 71% sensitivity and 63% specificity for predicting AKI [89]. The predictive ability of PLR was also demonstrated in a study by Sun et al. including also non-diabetic patients with ST-elevation myocardial infarction undergoing percutaneous coronary intervention (OR 1.432 (1.205–1.816), *p* = 0.031) [90]. Sun et al. demonstrated that a PLR of 127.5 or higher had 76.8% sensitivity and 69.2% specificity to predict AKI [90].

In the setting of cardiac surgery, Parlar et al. also demonstrated that an elevated preoperative PLR was associated with early postoperative AKI [OR 1.06 (1.01–1.10), *p* = 0.01] [66]. The cut-off value to predict AKI determined in this study was 136.85, which demonstrated a sensitivity of 71.0% and specificity of 70.7% [66].

Interestingly, in a retrospective cohort of 10,859 critically ill AKI patients, Zheng et al. identified that both low and high PLRs were associated with an increased mortality risk [PLR < 90 HR 1.25 (1.12–1.39), *p* < 0.001; PLR > 311 HR 1.19 (1.08–1.31), *p* < 0.001] [91]. A lower PLR could result from the presence of thrombocytopenia which is also correlated with a worse prognosis in critically ill patients [91].

While promising, the PLR needs to analyze in further studies to confirm its validity as a diagnostic and prognostic marker.

## Strengths and limitations

The complete blood count could be a useful tool to estimate the risk of developing AKI and mortality. Several parameters and indirect inflammation markers have been studied over the years with encouraging results.

Although some studies defined AKI with different criteria, the majority of studies used the KDIGO classification. The availability, low cost, and efficacy of these markers are an important advantage. The evidence is significant and the results have been replicated in large populations and many different settings, as depicted in Table 3.

Nonetheless, some important limitations have to be addressed. First, the majority of these studies are of a retrospective and single-center design which can compromise the results. Second, investigation has established a correlation between these parameters, AKI and mortality, determining the poor prognosis in these patients which could reflect disease severity and has not been able to prove causality between these. Finally, standardized cut-off parameters remain to be determined.

Therefore, before generalizing the use of complete blood count to predict AKI, further studies must be performed to confirm and validate these results, in preference with prospective design, larger populations, and longer follow-up.

## Conclusion

AKI is a frequent complication in hospitalized patients with negative impact in the short and long term. It is crucial to identify easily available predictors of AKI to prevent, diagnose, and treat this complication, and to minimize the associated morbidity and mortality.

Research has developed new biomarkers, although their widespread use in clinical practice is still limited. The complete blood count could be useful to estimate the risk of developing AKI and mortality. Anemia, leucopenia, leucocytosis, and thrombocytopenia can estimate illness severity. NLR, NLPR, and PLR are simple and straightforward markers calculated from routine blood analysis, which have proven to be effective in predicting AKI and outcomes in multiple settings.

## Data Availability

Not applicable.
